# Efficacy and safety of sugammadex for neuromuscular blockade reversal in pediatric patients: an updated meta-analysis of randomized controlled trials with trial sequential analysis

**DOI:** 10.1186/s12887-022-03288-0

**Published:** 2022-05-19

**Authors:** Bingchen Lang, Lu Han, Linan Zeng, Qianqian Zhang, Shouming Chen, Liang Huang, Zhijun Jia, Qin Yu, Lingli Zhang

**Affiliations:** 1 Department of Pharmacy, West China Second University Hospital, Sichuan University, Chengdu, China; 2 Evidence-Based Pharmacy Center, West China Second University Hospital, Sichuan University, Chengdu, 610041 China; 3 Key Laboratory of Birth Defects and Related Diseases of Women and Children, Ministry of Education, Sichuan University, Chengdu, China; 4 Department of Anesthesiology, Chengdu Women’s and Children’s Central Hospital, Chengdu, China; 5 Department of Anesthesiology, West China Second University Hospital, Sichuan University, Chengdu, China; 6 Department of Biopharmaceutics, West China School of Pharmacy, Sichuan University, Chengdu, China; 7 National Drug Clinical Trial Institute, West China Second University Hospital, Sichuan University, Chengdu, 610041 China

**Keywords:** Sugammadex, Children, Neuromuscular blockade, Acetylcholinesterase inhibitors, Meta-analysis

## Abstract

**Background:**

A recent survey revealed that extensive off-label use of sugammadex in pediatric anesthesia deserved particular attention. The present study with trial sequential analysis (TSA) aimed to evaluate the effects of sugammadex for antagonizing neuromuscular blockade (NMB) in pediatric patients, and to investigate whether the findings achieved the required information size to draw conclusions.

**Methods:**

PubMed, Embase, Cochrane Library and China National Knowledge Infrastructure (CNKI) were searched from inception to April 2021. All randomized controlled trials used sugammadex as reversal agent in pediatric patients were enrolled. Time from NMB reversal to recovery of the train-of-four ratio (TOFr) to 0.9 and extubation time were considered as co-primary outcomes, and incidences of adverse events were considered as secondary outcomes. Grading of Recommendations Assessment, Development, and Evaluation (GRADE) system was used to rate the quality of evidences.

**Results:**

Data from 18 studies involving 1,065 pediatric patients were acquired. The results revealed that use of sugammadex was associated with shorter duration from administration of reversal agents to TOFr > 0.9 (MD = -14.42, with 95% CI [-17.08, -11.75]) and shorter interval from reversal from NMB to extubation (MD = -13.98, with 95% CI [-16.70, -11.26]) compared to control groups. TSA also indicated that the current sample sizes were sufficient with unnecessary further trials. Analysis of secondary outcomes indicated that administration of sugammadex was associated with less incidence of postoperative nausea and vomiting (PONV), bradycardia, and dry mouth compared to control groups.

**Conclusion:**

Considering of satisfactory and rapid neuromuscular blockade reversal with low incidences of adverse events, sugammadex might be considered as the preferred option for children in clinical anesthesia practice compared to acetylcholinesterase inhibitors. However, overall low-quality evidences in present study rated by GRADE system indicated that superiority of sugammadex employed in pediatric patients needs to be confirmed by more studies with high quality and large sample size in future.

**Supplementary Information:**

The online version contains supplementary material available at 10.1186/s12887-022-03288-0.

## Introduction

The wide use of neuromuscular blocking agents (NMBAs) has revolutionized clinical anesthetic practice. It produces rapid profound skeletal muscle relaxation, provides convenient tracheal intubation, and improves surgical operating conditions [1]. However, it exposes patients to the risks of residual neuromuscular blockade (NMB) including postoperative pulmonary diseases and respiratory complications (pulmonary atelectasis, decreased oxygen saturation, upper airway obstruction) [2, 3], and leads to reintubation and excess length of stay.

Before sugammadex arrived on the scene, neostigmine, one of acetylcholinesterase inhibitors, was routinely used to competitively reverse the blockade of non-depolarizing muscle relaxants (e.g. rocuronium). However, application of neostigmine gives rise to various negative effects including bradycardia, hypersalivation, and bronchoconstriction. Therefore, to antagonize muscarinic side-effects, anticholinergics (e.g. atropine, glycopyrrolate) are recommended to be administered in a mixture with neostigmine. In addition, due to an absence of ability to reverse the blockade from rocuronium immediately, use of neostigmine may increase risks of post-operative residual neuromuscular block [4–6].

As the first non-competitive antagonist for the reversal of NMB, sugammadex, a modified γ-cyclodextrin, features its unique mechanism different from the mechanism of acetylcholinesterase inhibitors. It rapidly encapsulates rocuronium or vecuronium by one-to-one molecular binding, provides fast and predictable reversal effects of NMB, and decreases the incidence of residual block efficiently [7–9]. Since the first-in-man clinical research in 2005 [10], sugammadex has been used extensively in surgical practice for adult patients in recent years [11]. Simultaneously, although the drug package insert evidently declares that “the safety and efficacy of sugammadex in pediatric patients have not been established” [12], according to recent studies [13, 14] and a latest survey [15], this novel agent has been frequently used in pediatric anesthesia, especially among anesthesiologists with fewer years of practice.

In an effort to evaluate the effects of sugammadex on pediatric patients, Won et al*.* [16] and Liu et al*.* [17] conducted the relevant meta-analyses, and both of two studies demonstrated its effective and rapid profiles in reversing NMB. However, authors described that included studies still lacking sufficient information. It also requires more evidences to draw the reliable conclusions.

Therefore, on the basis of combining the latest evidences in various regions, we conducted the present updated meta-analysis by reviewing RCTs (randomized controlled trials) to compare the efficacy and safety between sugammadex and acetylcholinesterase inhibitors, so as to accumulate more information about the use of sugammadex for antagonizing rocuronium-induced NMB in pediatric patients. And the trial sequential analysis (TSA) was also performed to determine whether the findings achieved the required information size to draw the conclusions.

The present meta-analysis was performed in accordance with the recommendations in the Preferred Reporting Items for Systematic Reviews and Meta-Analyses (PRISMA) statement [18] and the guidelines described in the Cochrane Handbook.

## Methods

### Search strategy

Two independent authors (BL and QZ) searched PubMed, Embase, Cochrane Library, and CNKI (China National Knowledge Infrastructure) databases up to April 24, 2021. Moreover, we considered potentially useful studies in Google Scholar as additional sources of information. The search terms we used included infant, child, adolescent, sugammadex, org 25,969, bridion and randomized controlled trial (Appendix S1). Only human studies were involved, and there were no restrictions of language.

### Eligibility criteria

The studies meeting the following conditions were selected for further analysis:

#### Participants

The patients were the pediatric patients (< 18 years old) who experienced different surgical and diagnostic procedures.

#### Intervention and comparison

Using sugammadex (regardless of administration doses) versus acetylcholinesterase inhibitors or placebo as reversal agent.

#### Outcome measures

Given that rapid recovery from NMB to a train-of-four (TOF) ratio of 0.9, short duration from reversal injection to extubation, and limited adverse effects were considered as the ideal characteristics of a reversal agent [19], the co-primary outcomes were as follows: (a) time from NMB reversal to recovery of the TOF ratio to 0.9, (b) extubation time. And incidences of adverse events were considered as secondary outcomes.

#### Study design

Randomized controlled trials with no language limitations.

### Data extraction, and assessment of the risk of bias

Two authors (BL and QZ) conducted the data extraction and identified quality and eligibility of studies. After removing the duplicates from different databases, those obviously irrelevant records were excluded by titles and abstracts screening. The full texts of the remaining studies were obtained and perused. To collect the general characteristics of enrolled studies, a table was designed and filled by us (Table 1). The risk of bias in RCTs was evaluated by the Cochrane risk of bias tool [20], using the following domains: random sequence generation (generation of the randomization sequence), allocation concealment, blinding of outcome assessment, incomplete outcome data, and selective reporting. All articles could have the following domain classifications: high risk of bias, low risk of bias, uncertain risk (without information for judgment). Any disagreements were resolved by consensus through discussion.

**Table 1:** The general characteristics of the enrolled studies

Study (Reference)	Year	Region	Type of surgery	Patient age range & ASA status	Patients enrolled (Gender: F/M, n)	Dosage of NMBA	Intervention (n)	Comparison (n)	Time of reversal agents administration	Outcomes
Plaud B [21]	2009	Multicenter (France, UK, Finland and Germany)	Surgery in supine position	28 d-17 y (ASA I-II)	31/32, 62	Rocuronium 0.6 mg/kg	Sugammadex (0.5, 1.0, 2.0, or 4.0 mg/kg) (*n* = 47)	Placebo (*n* = 11)	Reappearance of T2	I, III
Veiga RG [22]	2011	Spain	Elective surgery	2–9 y (Not mentioned)	24	Rocuronium 0.45 mg/kg	Sugammadex 2.0 mg/kg (*n* = 14)	Neostigmine 5 mcg/kg + atropine 2.5 mcg/kg(*n* = 10)	Reappearance of T2 three timess	I
Alvarez- Gomez JA [23]	2012	Multicenter	Not mentioned	2–11 y (Not mentioned)	96	Rocuronium 0.6 mg/kg	Sugammadex 4.0 mg/kg (*n* = 49)	Neostigmine 50 mcg/kg + atropine 25 mcg/kg (*n* = 47)	Post-titanic count > 2	I-III
Gaona D [24]	2012	Multicenter	Short length surgery	2–11 y (Not mentioned)	30	Rocuronium 0.6 mg/kg	Sugammadex 4.0 mg/kg (*n* = 15)	Neostigmine 50 mcg/kg + atropine 25 mcg/kg (*n* = 15)	Post-titanic count < 2–3 in Sugammadex group; Post-titanic count > 2–3 in Control group	I-III
Kara T [25]	2014	Turkey	Elective lower abdominal/urogenital procedures	2–12 y (ASA I)	80	Rocuronium 0.6 mg/kg	Sugammadex 2.0 mg/kg (*n* = 40)	Neostigmine 30 mcg/kg + atropine 10 mcg/kg (*n* = 40)	Reappearance of T2	I-III
Ozgün C [26]	2014	Turkey	Ear nose and throat surgery	2–12 y (ASA I)	29/31, 60	Rocuronium 0.6 mg/kg	Sugammadex 2.0 mg/kg (*n* = 30)	Neostigmine 60 mcg/kg + atropine 20 mcg/kg (*n* = 30)	Reappearance of T2	I, III
Ghoneim AA [27]	2015	Egypt	Elective craniotomy for posterior fossa tumor excision	7–18 y (ASA I-III)	20/20,40	Rocuronium 0.6 mg/kg	Sugammadex 4.0 mg/kg (*n* = 20)	Neostigmine 40 mcg/kg + atropine 20 mcg/kg (*n* = 20)	Reappearance of T2	I, III
El sayed M [28]	2016	Egypt	Outpatient tonsillectomy	2–10 y (Not mentioned)	37/33,70	Rocuronium 0.6 mg/kg	Sugammadex 2.0 mg/kg (*n* = 35)	Neostigmine 50 mcg/kg + atropine 10 mcg/kg (*n* = 35)	Reappearance of T2	I-III
Güzelce D [29]	2016	Turkey	Lower urinary tract surgery and inguinal hernia	2–17 y (ASA I)	37	Rocuronium 0.6 mg/kg	Sugammadex 2.0 mg/kg (*n* = 16)	Neostigmine 50 mcg/kg + atropine 20 mcg/kg (*n* = 21)	Reappearance of T2	I-III
Mohamad Zaini RH [30]	2016	Malaysia	Not mentioned	2–18 y (ASA I-II)	21/59,80	Rocuronium 0.6 mg/kg	Sugammadex 2.0 mg/kg (*n* = 40)	Neostigmine 50 mcg/kg + atropine 20 mcg/kg (*n* = 40)	Reappearance of T2	I-III
Ammar AS [31]	2017	Egypt	Lower abdominal surgery	2–10 y (ASA I-II)	22/38, 60	Rocuronium 0.6 mg/kg	Sugammadex 4.0 mg/kg (*n* = 30)	Neostigmine 0.35 mg/kg + atropine 0.02 mg/kg (*n* = 30)	Post-titanic count of 1–2 in Sugammadex group; Reappearance of T2 in Control group	I-III
Korkmaz MO [32]	2019	Turkey	Adenotonsillectomy	2–13 y (Not mentioned)	30/40, 70	Rocuronium 0.6 mg/kg	Sugammadex 2.0 mg/kg (*n* = 35)	Neostigmine 20 mcg/kg + atropine 10 mcg/kg (*n* = 35)	Reappearance of T2	II, III
An J [33]	2019	Korea	Entropion surgery	2–11 y (ASA I-II)	26/34, 60	Rocuronium 0.6 mg/kg	Sugammadex 2.0 mg/kg (*n* = 30)	Pyridostigmine 0.2 mg/kg + glycopyrrolate 0.01 mg/kg (*n* = 30)	TOF ratio ≥ 0.1	I-III
Hussein AA [34]	2020	Egypt	Outpatient surgical procedures	2–18 y (ASA I-II)	43/37, 80	Rocuronium 0.6 mg/kg	Sugammadex 2.0 mg/kg (*n* = 40)	Neostigmine 30 mcg/kg + atropine 20 mcg/kg (*n* = 40)	Reappearance of T2	I-III
Li XB [35]	2020	China	Elective cardiac surgery	2–6 y (ASA II-III)	26/34, 60	Rocuronium 0.6 mg/kg	Sugammadex 4.0 mg/kg (*n* = 30)	Placebo (*n* = 30)	TOF ratio = 0 and PTC = 1–2	I-III
Li L [36]	2020	China	Cardiac surgery	1–6 y (ASA II-III)	34/26, 60	Rocuronium 0.6 mg/kg	Sugammadex 4.0 mg/kg (*n* = 30)	Neostigmine 30 mcg/kg + atropine 15 mcg/kg (*n* = 30)	TOF ratio ≥ 0.25	I-III
Hu J [37]	2020	China	Laparoscopic inguinal hernia repair	6 m-7 y (ASA I-II)	8/32, 40	Rocuronium 0.6 mg/kg	Sugammadex 2.0 mg/kg (*n* = 20)	Neostigmine 0.05 mg/kg + atropine 0.01 mg/kg (*n* = 20)	Reappearance of T2	I-III
Jiang Y [38]s	2020	China	Elective tonsillectomy	3–6 y (ASA I-II)	60	Rocuronium 0.6 mg/kg	Sugammadex 2.0 mg/kg (*n* = 30)	Neostigmine 40 mcg/kg + atropine 20 mcg/kg (*n* = 30)	Reappearance of T2	I-III

I—Time interval from administration of reversal agents to train-of-four ratio (TOFr, T4/T1) > 0.9; II—Extubation time; III—Adverse effectss

*NMBA* neuromuscular blocking agent, *ASA* American Society of Anesthesiologist physical status, *TOF* train-of-four, *PTC* post tetanic count

### Grading the quality of evidence

Assessment of quality of evidence and strength of recommendations was conducted by using the Grading of Recommendations Assessment, Development, and Evaluation (GRADE) methodology [39]. The quality of outcomes was independently assessed by two authors (BL and QZ). On the basis of risk of bias, inconsistency, indirectness, imprecision, and publication bias, the quality was classified as high, moderate, low, or very low. The GRADE profiler (version 3.6) software was used.

### Statistical analysis

Statistical analyses were performed by using Review Manager software (Version 5.3.3, the Cochrane Collaboration 2014, the Nordic Cochrane Centre). Mean difference (MD) with 95% confidence interval (CI) were used to estimate continuous variables, and risk ratio (RR) with 95% confidence interval (CI) and the Mantel–Haenszel method (fixed or random models) were used to analyze dichotomous data. The I-squared (*I*^*2*^) test was chosen to weigh the impact of heterogeneity on the results. If significant heterogeneity (present at *I*^*2*^ > 50%) existed, the sensitivity analysis was performed by omitting each study individually, and the random effects model was chosen; otherwise, the fixed-effects model was chosen. Publication bias were evaluated by using Begg's test and Egger’s test if the number of included studies exceeds 10. Evaluation was performed using version 1.2.4 of the metabias program, Stata/MP 12.0 for Windows (StataCorp LP, 4905 Lakeway Drive, College Station, TX 77,845, USA). A *P* value < 0.05 was considered statistically significant.

Sparse data and the repeated significance testing with new studies updating may lead to type-1 errors (false-positive outcomes) and type-2 errors (false-negative outcomes) of meta-analyses. To eliminate the risks from type-1 and type-2 errors, Trial sequential analysis (TSA), which can adjust the statistical threshold by controlling *P* value and widening confidence intervals, was performed by us. TSA can estimate the required information size (RIS) and trial sequential monitoring boundaries. The cumulative Z curve entering the futility area or crossing the trial sequential monitoring boundary may indicate that the present evidences of intervention effects are at a sufficient level, and further trials will be unnecessary. Otherwise, evidences are insufficient to draw the conclusion if Z curve does not cross any boundaries or reach the RIS [40]. And the TSA was performed using Trial Sequential Analysis Viewer Software (version 0.9.5.10 beta; http://www.ctu.dk/tsa).

## Results

### Literature search results

**Fig. 1:**
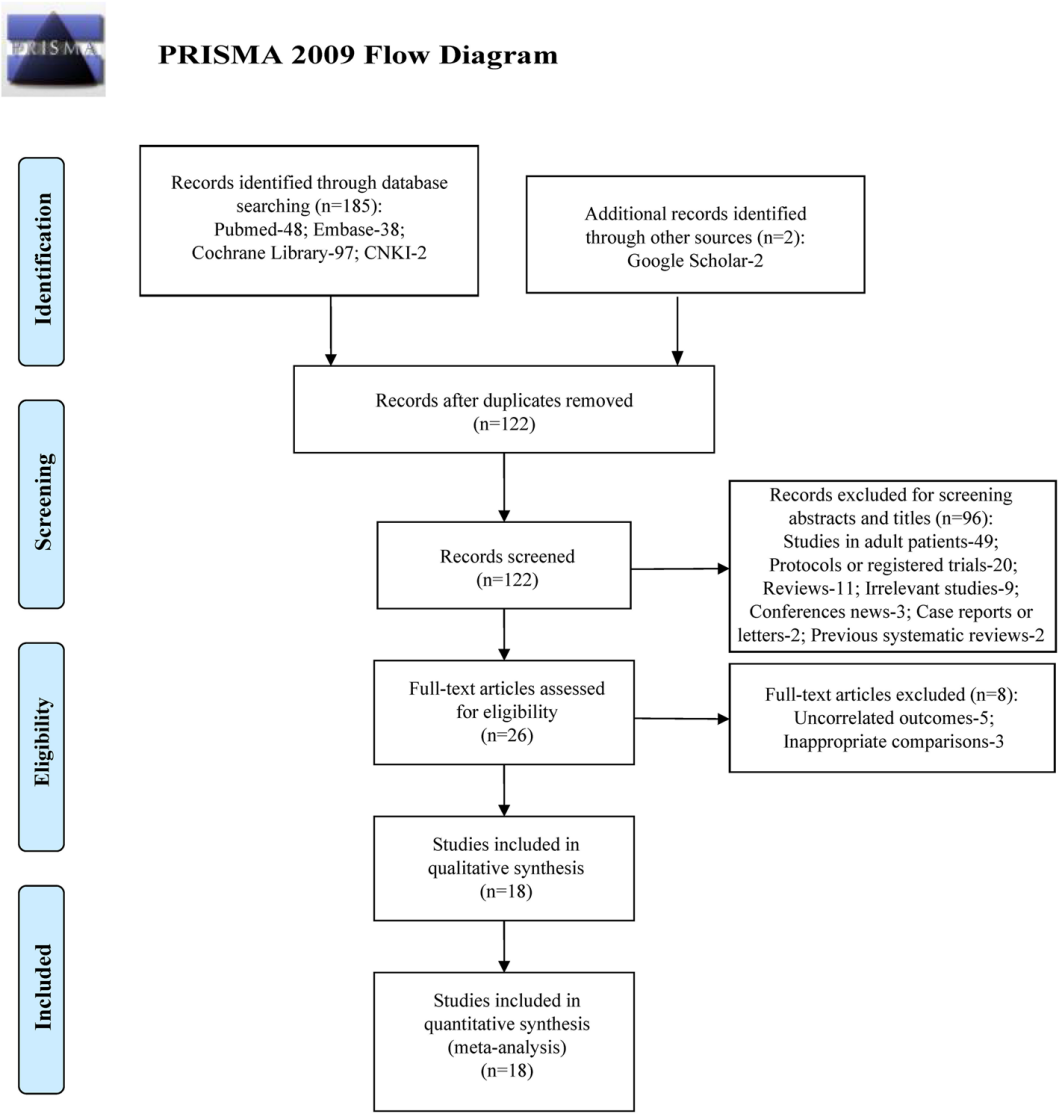
Flow chart of literature screening and the selection process

After screening in databases and additional sources of information, a total of 187 relevant items were identified initially. 65 duplicate records were removed, and 96 records were excluded by titles and abstracts reviewing. In these 96 excluded items, 49 were studies conducted in adult patients, 20 were protocols or registered trials, 11 were reviews, 9 were irrelevant studies, 3 were conferences news, 2 were case reports or letters, 2 were previous systematic reviews published in 2016 and in 2017. And then 8 items were excluded by full-text screening, five of them reported the uncorrelated outcomes, and three of them were owing to the inappropriate comparisons. Eventually, 18 studies were chosen in consequent analysis [21–38]. The process of literatures identification is described in PRISMA flowchart (Fig. 1).

### Basic characteristics of enrolled studies

The enrolled studies were published from 2009 to 2020, and a total of 1,065 eligible pediatric patients (ages ranged from 7 days to 18 years) were included in analysis. The outcome “time interval from administration of reversal agents to train-of-four ratio” was reported in 17 studies [21–31, 33–38], and the outcome “extubation time” was reported in 14 studies [23–25, 28–38]. 0.6 mg/kg rocuronium was given in all patients except patients in Veiga RG et al*.* study [22] (Rocuronium 0.45 mg/kg). And most of studies focused on evaluation in sugammadex versus combination of acetylcholinesterase inhibitors and anticholinergics, only two studies compared sugammadex with placebo. The main characteristics of all enrolled studies were summarized in Table 1.

### Risk of bias assessment

We used Cochrane Collaboration’s risk of bias tool to evaluate the validity and quality of these enrolled studies [20]. In random sequence generation domain, 12 studies had low risk [21, 25, 27–34, 36, 37], and 6 studies had unclear risk [22–24, 26, 35, 38]. In allocation concealment domain, 6 studies had low risk of bias [21, 26, 29, 30, 32, 34], and 12 studies had unclear risk [22–25, 27, 28, 31, 33, 35–38]. Ten studies had low risk of bias [21, 24, 26, 28, 30–34, 36] and rest of studies had unclear risk of bias [22, 23, 25, 27, 29, 35, 37, 38] in blinding of participants and personnel domain. One study had a high risk of bias [28], 9 studies had low risk of bias [21, 24, 26, 30–34, 36], and 8 studies had unclear risk [22, 23, 25, 27, 29, 35, 37, 38] in blinding of outcome assessment domain. Sixteen studies had low risk of bias in incomplete outcome data [21, 23, 25–38] and rest of studies had unclear risk of bias [22, 24]. In selective reporting domain, 16 studies had low risk [21, 23–31, 33–38], and two studies had unclear risk of bias [22, 32].

### Primary outcome 1: time interval from administration of reversal agents to train-of-four ratio (TOFr) > 0.9

**Fig. 2:**
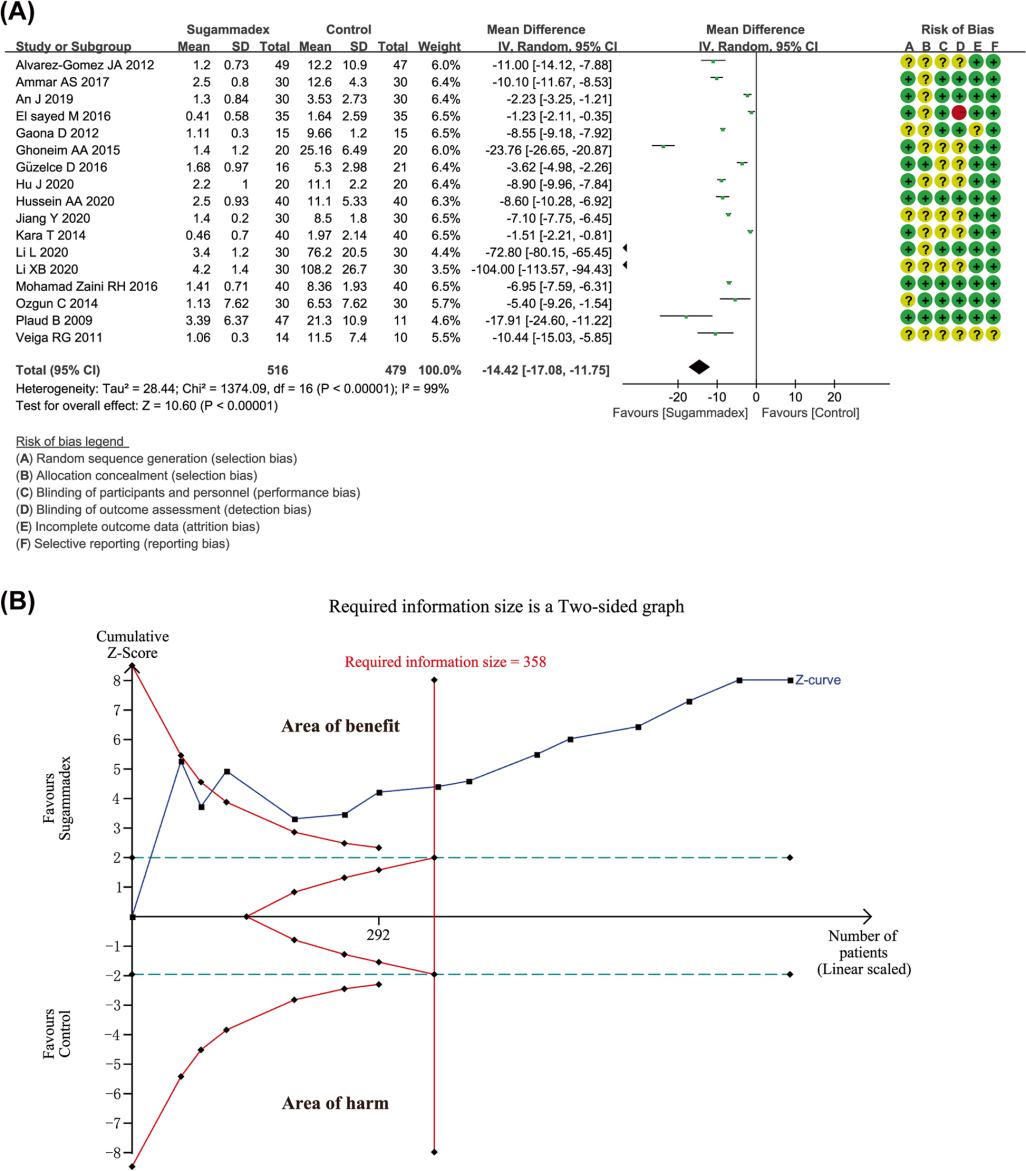
**A** Forest plot depicting the meta-analysis for the outcome “time interval from administration of reversal agents to train-of-four ratio (TOFr) > 0.9” for sugammadex versus controls; **B** Trial sequential analysis for the outcome “time interval from administration of reversal agents to train-of-four ratio (TOFr) > 0.9”. Notes: Green + dot, low risk of bias; yellow ? dot, unclear risk of bias; red—dot, high risk of bias. (Abbreviations: CI, Confidence interval)

Seventeen studies including 995 pediatric patients described the time from NMB reversal to recovery of the TOF ratio to 0.9. The *I*^2^ of 99% indicated that substantial heterogeneity was existed, but the source could not be attributed clearly to one particular study by sensitivity analysis; thus, the random effects model was used. According to present analysis with larger sample size, the use of sugammadex was associated with significantly shorter duration from administration of reversal agents to TOFr > 0.9 compared to traditional acetylcholinesterase inhibitors or placebo (MD -14.42 with 95% CI [-17.08, -11.75], *P* < 0.00001, *I*^2^ = 99%) (Fig. 2A). Publication bias was detected in analysis by both Begg's test (*P* = 0.001) and Egger's test (*P* = 0.000) (Fig. 4A). In order to estimate and adjust for the number and outcomes of missing studies, we performed Duval's trim and fill method [41] by using version 1.0.5 of the metatrim program, Stata/MP 12.0 for Windows (StataCorp LP, 4905 Lakeway Drive, College Station, TX 77,845, USA). The trim-and-fill method showed no trimming performed and data unchanged. The information about trim and fill procedure was provided in Appendix S2. The outcome of TSA indicated that the cumulative Z curves crossed the conventional boundary, trial sequential monitoring boundary, and also the required information size (calculated as 358). It revealed that the sample size of patients was enough, and further studies would be unlikely to change the conclusion (Fig. 2B). According to GRADE summary of findings table, the quality of evidence for this outcome was low. It might be resulted from inconsistency (*I*^2^ > 50%) and existed publication bias (Table S1).

### Primary outcome 2: extubation time

**Fig. 3:**
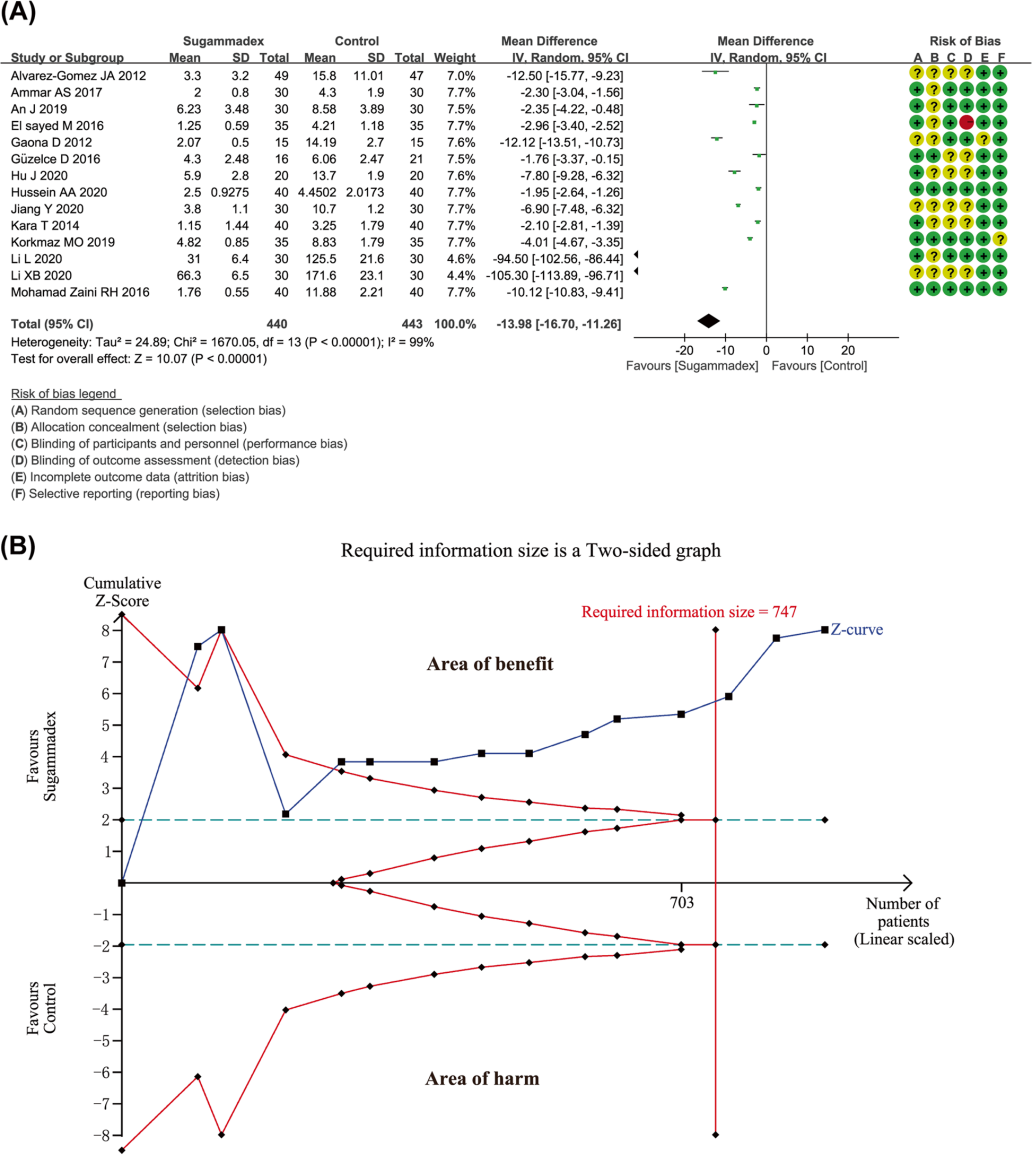
**A** Forest plot depicting the meta-analysis for the outcome “extubation time” for sugammadex versus controls; **B** Trial sequential analysis for the outcome “extubation time”. Notes: Green + dot, low risk of bias; yellow ? dot, unclear risk of bias; red—dot, high risk of bias. (Abbreviations: CI, Confidence interval)

**Fig. 4:**
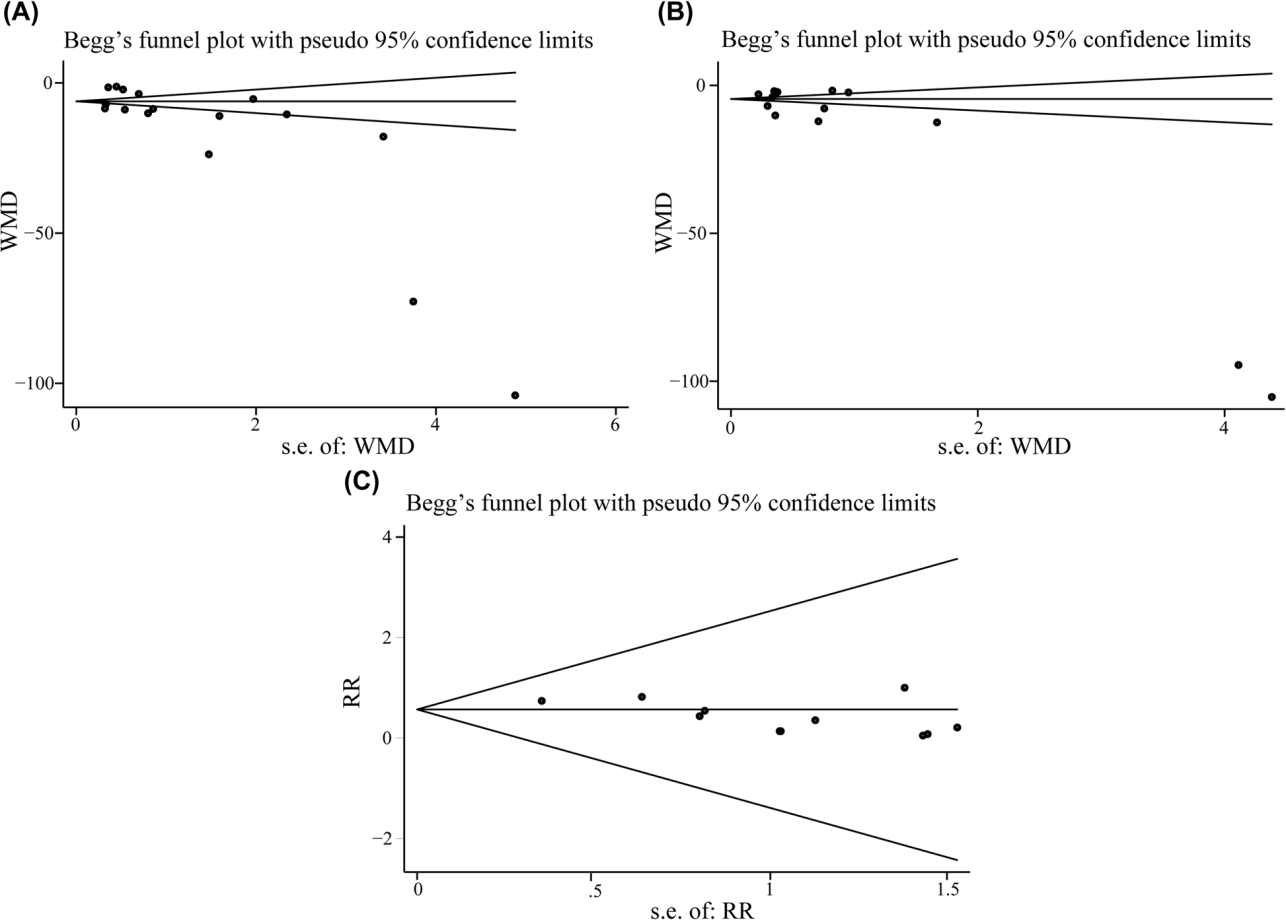
Funnel plots of effect estimates for the outcomes. **A** time interval from administration of reversal agents to train-of-four ratio (TOFr) > 0.9; **B** extubation time; **C** the incidence of postoperative nausea and vomiting (PONV). (Abbreviations: WMD, weighted mean difference; RR, risk ratio)

A total of 14 studies involving 883 pediatric patients reported the duration from NMB reversal to extubation. By the same token, *I*^2^ of 99% existed the significant heterogeneity. However, all attempts to reduce the value of *I*^2^ to below 50% by excluding one single study were not successful in sensitivity analysis, therefore, the random effects model was used by us. The use of sugammadex was associated with shorter interval from reversal from NMB to extubation compared to acetylcholinesterase inhibitors or placebo (MD -13.98 with 95% CI [-16.70, -11.26], *P* < 0.00001, *I*^2^ = 99%) (Fig. 3A). However, results from Begg's test (*P* = 0.002) and Egger’s test (*P* = 0.000) indicated that publication bias was existed in the analysis (Fig. 4B). Duval's trim and fill method was conducted, and results showed no trimming performed and data unchanged. The information about trim and fill procedure was provided in Appendix S2. The result from TSA indicated that with a required information size of 747, firm evidence was in place in favor of sugammadex (Fig. 3B). The GRADE summary of findings table indicated that quality of evidence for present outcome was low. Inconsistency (*I*^2^ > 50%) and publication bias may be considered as main factors (Table S1).

### Secondary outcomes

Adverse effects including postoperative nausea and vomiting (PONV), bradycardia, pain, spasm, dry mouth, apnea, and oxygen desaturation were considered as our secondary outcomes. The results indicated that use of sugammadex was associated with significantly lower incidence of PONV (RR = 0.30; 95%CI: 0.20 to 0.46), bradycardia (RR = 0.09; 95%CI: 0.02 to 0.46), and dry mouth (RR = 0.14; 95%CI: 0.05 to 0.38) compared to acetylcholinesterase inhibitors or placebo. For other adverse effects, no significant differences were found between the two groups. The results of publication bias were (*P* = 0.088, Begg’s test and *P* = 0.004, Egger’s test) (Fig. 4C), however, the trim-and-fill method to adjust for funnel plot asymmetry showed no trimming performed and data unchanged. Owing to absence of statistical heterogeneity (*I*^2^ < 50%) in secondary outcomes, the fixed-effects model was used. The details of secondary outcomes were demonstrated in Table 2. The results from GRADE summary of table revealed that quality of evidence for most of secondary outcomes was low and imprecision (lack of events number) was served as the main reason. The details were provided in Table S1.

**Table 2:** Secondary outcomes

Adverse effects	Number of studies (Reference no.)	Patients in Sugammadex group (Incidence, %)	Patients in Control group (Incidence, %)	*I*^2^ (%)	Risk ratio with [95% CI]	*P* value
PONV	13 (23,25,28,29,32–40)	33/431 (7.66%)	69/393 (17.56%)	21	0.30 [0.20, 0.46]	< 0.00001*
Bradycardia	4 (25,26,33,40)	0/124 (0%)	15/122 (12.30%)	0	0.09 [0.02, 0.46]	0.004*
Pain	2 (23,39)	8/67 (11.94%)	5/31 (16.13%)	0	1.21 [0.46, 3.17]	0.70
Bronchospasm/ Laryngospasm	3 (25,28,34)	1/114 (0.88%)	4/112 (3.57%)	0	0.45 [0.10, 1.96]	0.29
Dry mouth	2 (33,35)	3/60 (5%)	25/60 (41.67%)	0	0.14 [0.05, 0.38]	0.0001*
Apnea	2 (34,40)	0/65 (0%)	2/65 (3.08%)	0	0.33 [0.04, 3.12]	0.34
Oxygen desaturation	3 (34,35,38)	3/95 (3.16%)	8/95 (8.42%)	0	0.41 [0.12, 1.37]	0.15

^*^Significant difference between groups (*P* < 0.05)

*PONV* postoperative nausea and vomiting, *CI* confidence intervals

## Discussion

The meta-analyses conducted by Won et al*.* [16] and Liu et al*.* [17] included RCTs published during 2016–2017 and demonstrated the superiority of sugammadex in providing rapid recovery in children. However, limited sample size (253 patients and 575 patients individually) of the two studies and increasing clinical applications of sugammadex in recent years prompted us to update the research.

Our present study evaluated a total of 18 RCTs enrolled over 1000 pediatric patients. The results indicated that administration of sugammadex in children was associated with shorter duration from administration of reversal agents to TOFr > 0.9 and shorter interval from reversal from NMB to extubation compared to acetylcholinesterase inhibitors or placebo. It confirmed and strengthened the findings of previous meta-analyses. And TSA results from our present study about the co-primary outcomes indicated that the present evidences of anticipated intervention effects were sufficient.

As one of main adverse effects appeared in post-anaesthesia care units (PACU), PONV after general anaesthesia may be resulted from multiple causative factors, such as inhalational anaesthesia and perioperative opioids use [42]. The study conducted by Liu et al*.* [17] described no difference in incidence of nausea and vomiting between sugammadex group and control group. However, the present study with a larger sample size demonstrated that the application of sugammadex was associated with significantly lower incidence of PONV in pediatric patients compared to control group.

According to previous retrospective analysis and review [14, 43], bradycardia, one of significant adverse effects of NMB reversal agents, was found more commonly in neostigmine patients than in sugammadex patients. The results of our present study suggested that incidence of both bradycardia and dry mouth was significant lower in sugammadex patients, and no difference was found in occurrence of pain, bronchospasm, laryngospasm, apnea and oxygen desaturation between two groups. Regrettably, even though we performed a thorough search including several international and one Chinese database, the sample size of most secondary outcomes was still limited, and it was insufficient to draw reliable conclusions.

Another limitation from our present study was the widespread low quality in outcomes exhibited by GRADE approach evaluation, which resulted from publication bias, inconsistency (high heterogeneity) and imprecision (lack of events number). The results of Begg's test and Egger's test indicated that publication bias were existed in several outcomes. However, the trim-and-fill method to adjust for funnel plot asymmetry revealed no trimming performed and data unchanged. Actually, publication bias should be considered as one major difficulty in systematic reviews. The researches with statistically significant results were tend to be the ones accepted for publication rather than studies with inconclusive outcomes or with no obvious treatment effects [44]. Therefore, the review of published studies might be identified as a biased selection of the researches, and sometimes the problems from publication bias were inevitable. Therefore, to overcome the problems, we conducted a thorough search for grey literature from websites “http://www.greylit.org/” and “http://greyguide.isti.cnr.it/” by using key terms “sugammadex” or “bridion” or “25,969” or “361LPM2T56” (Accessed 6 April 2022). However, no results were found. In addition, the attempts to reduce high heterogeneity by excluding one single study were failed in sensitivity analysis, and it led us to use random effects models for meta-analysis.

## Conclusion

Although detected heterogeneity was considerable in primary outcomes, the results of present study demonstrated that the use of sugammadex was associated with more rapid reversal of rocuronium-induced neuromuscular blockade when compared with control groups. And TSA provided firm evidence in favor of sugammadex for primary outcomes. However, overall low-quality evidences evaluated by GRADE system demonstrated that superiority of sugammadex in providing adequate efficacy and safety of NMB reversal in children needs to be confirmed by more studies with high quality and large sample size in future.

## Supplementary Information

**Additional file 1.**

**Additional file 2.**

**Additional file 3: Table S1.** GRADE summary of findings table.

## Data Availability

All data generated or analysed during this study are included in this published article [and its supplementary information files].

## Abbreviations

TSA: Trial sequential analysis
NMB: Neuromuscular blockade
TOFr: Train-of-four ratio
GRADE: Grading of recommendations assessment, development, and evaluation
PONV: Postoperative nausea and vomiting
NMBAs: Neuromuscular blocking agents
RCTs: Randomized controlled trials
CNKI: China National Knowledge Infrastructure
MD: Mean difference
CI: Confidence interval
RR: Risk ratio
RIS: Required information size
PACU: Post-anaesthesia care units
