# Supplementary material for: Efficacy and safety of sugammadex for neuromuscular blockade reversal in pediatric patients: an updated meta-analysis of randomized controlled trials with trial sequential analysis
Source: BMC Pediatr. 2022 May 19;22:295. doi: 10.1186/s12887-022-03288-0 (PMC9118813; doi:10.1186/s12887-022-03288-0)
Supplement: Supplementary file 1 — Additional file 1. [file 12887_2022_3288_MOESM1_ESM.docx]

2021.4.24

**Pubmed**

| **Search** | **Query** |
| --- | --- |
| #1 | Infant [mesh] |
| #2 | newborn* [tiab] or neonat* [tiab] or infant* [tiab] or infancy [tiab] or baby [tiab] or babies [tiab] or toddler* [tiab] |
| #3 | #1 OR #2 |
| #4 | Child [mesh] |
| #5 | Pediatrics [mesh] |
| #6 | p?ediatric* [tiab] or child* [tiab] or kindergar* [tiab] or preschool* [tiab] or kid [tiab] or kids [tiab] or schoolchild* [tiab] or “school age” [tiab] or schoolage [tiab] or preteen* [tiab] or youth* [tiab] or prepubescent* [tiab] |
| #7 | #4 OR #5 OR #6 |
| #8 | Adolescent [mesh] |
| #9 | adolesc* [tiab] or teen* [tiab] or youth* [tiab] or underage* [tiab] or “under age*” [tiab] or minor* [tiab] or juvenile* [tiab] or pubert* [tiab] or pubescen* [tiab] or “young people*” [tiab] or “young person*” [tiab] or “young adult*” [tiab] |
| #10 | #8 OR #9 |
| #11 | #3 OR #7 OR #10 |
| #12 | "Sugammadex" [nm] OR "Sugammadex" [all] OR "sugammadex" [all] OR "bridion" [all] OR "25969" [all] OR "361LPM2T56" [rn] |
| #13 | (randomized controlled trial [pt] OR controlled clinical trial [pt] OR randomized [tiab] OR placebo [tiab] OR clinical trials as topic [mesh: noexp] OR randomly [tiab] OR trial [ti]) NOT (animals [mh] NOT humans [mh]) |
| #14 | #11 AND #12 AND #13 **(Items found: 48)** |

**Embase**

| **Search** | **Query** |
| --- | --- |
| #1 | 'Infant'/exp |
| #2 | (newborn* or neonat* or infant* or infancy or baby or babies or toddler*):ab,ti |
| #3 | #1 OR #2 |
| #4 | 'child'/exp |
| #5 | 'pediatrics'/exp |
| #6 | (paediatric*or pediatric* or child* or kindergar* or preschool* or kid or kids or schoolchild* or 'school age' or schoolage or preteen* or youth* or prepubescent*):ab,ti |
| #7 | #4 OR #5 OR #6 |
| #8 | 'adolescent'/exp |
| #9 | (adolesc* or teen* or youth* or underage* or "under age*" or minor* or juvenile* or pubert* or pubescen* or "young people*" or "young person*" or "young adult*"):ab,ti |
| #10 | #8 or #9 |
| #11 | (#3 or #7 or #10) and [embase]/lim |
| #12 | (Sugammadex or bridion or 25969 or 361LPM2T56):ab,ti |
| #13 | #12 and [embase]/lim |
| #14 | 'randomized controlled trial'/exp |
| #15 | 'controlled clinical trial'/exp |
| #16 | 'randomization'/exp |
| #17 | 'double blind procedure'/exp |
| #18 | 'single blind procedure'/exp |
| #19 | random*:ab |
| #20 | trial*:ab |
| #21 | #14 OR #15 OR #16 OR #17 OR #18 OR #19 OR #20 |
| #22 | 'human'/exp |
| #23 | #21 AND #22 AND [embase]/lim |
| #24 | #11 AND #13 AND #23 **(Items found: 38)** |

**Cochrane Library**

| **Search** | **Query** |
| --- | --- |
| #1 | MeSH descriptor: [Infant] explode all trees |
| #2 | (newborn* or neonat* or infant* or infancy or baby or babies or toddler*):ti,ab,kw |
| #3 | #1 or #2 |
| #4 | MeSH descriptor: [Child] explode all trees |
| #5 | MeSH descriptor: [Pediatrics] explode all trees |
| #6 | (paediatric*or pediatric* or child* or kindergar* or preschool* or kid or kids or schoolchild* or 'school age' or schoolage or preteen* or youth* or prepubescent*):ti,ab,kw |
| #7 | #4 or #5 or #6 |
| #8 | MeSH descriptor: [Adolescent] explode all trees |
| #9 | (adolesc* or teen* or youth* or underage* or "under age*" or minor* or juvenile* or pubert* or pubescen* or "young people*" or "young person*" or "young adult*"):ti,ab,kw |
| #10 | #8 or #9 |
| #11 | #3 or #7 or #10 |
| #12 | (Sugammadex or bridion or 25969 or 361LPM2T56):ti,ab,kw |
| #13 | #11 and #12 **(Items found: 97 Trials)** |

**CNKI**

| **Search** | **Query** |
| --- | --- |
| #1 | (TI='舒更葡糖' OR KY='舒更葡糖') |
| #2 | (TI='Sugammadex' OR KY='Sugammadex') |
| #3 | #1 or #2 |
| #4 | (TI='儿童' OR KY='儿童') OR (TI='儿科' OR KY='儿科') OR (TI='小儿' OR KY='小儿') |
| #5 | #3 and #4 **(Items found: 2)** |
