# Supplementary material for: Efficacy and safety of sugammadex for neuromuscular blockade reversal in pediatric patients: an updated meta-analysis of randomized controlled trials with trial sequential analysis
Source: BMC Pediatr. 2022 May 19;22:295. doi: 10.1186/s12887-022-03288-0 (PMC9118813; doi:10.1186/s12887-022-03288-0)
Supplement: Supplementary file 2 — Additional file 2. [file 12887_2022_3288_MOESM2_ESM.docx]

**Outcome 1 Time interval from administration of reversal agents to train-of-four ratio (TOFr) > 0.9**

| Pooled 95% CI Asymptotic No. of

Method | Est Lower Upper z_value p_value studies

-------+----------------------------------------------------

Fixed | -1.955 -2.124 -1.787 -22.738 0.000 17

Random | -3.161 -3.955 -2.368 -7.813 0.000

Test for heterogeneity: Q= 326.641 on 16 degrees of freedom (p= 0.000)

Moment-based estimate of between studies variance = 2.529

Trimming estimator: Linear

Meta-analysis type: Random-effects model

iteration | estimate Tn # to trim diff

----------+--------------------------------------

1 | -3.161 75 0 153

2 | -3.161 75 0 0

Note: no trimming performed; data unchanged

**Outcome 2 Extubation time**

Meta-analysis

| Pooled 95% CI Asymptotic No. of

Method | Est Lower Upper z_value p_value studies

-------+----------------------------------------------------

Fixed | -2.042 -2.222 -1.861 -22.207 0.000 14

Random | -3.233 -4.127 -2.340 -7.092 0.000

Test for heterogeneity: Q= 298.130 on 13 degrees of freedom (p= 0.000)

Moment-based estimate of between studies variance = 2.693

Trimming estimator: Linear

Meta-analysis type: Random-effects model

iteration | estimate Tn # to trim diff

----------+--------------------------------------

1 | -3.233 44 0 105

2 | -3.233 44 0 0

Note: no trimming performed; data unchanged

**Outcome 3 Incidence of PONV**

Meta-analysis

| Pooled 95% CI Asymptotic No. of

Method | Est Lower Upper z_value p_value studies

-------+----------------------------------------------------

Fixed | 0.570 0.128 1.011 2.530 0.011 13

Random | 0.570 0.128 1.011 2.530 0.011

Test for heterogeneity: Q= 1.274 on 12 degrees of freedom (p= 1.000)

Moment-based estimate of between studies variance = 0.000

Trimming estimator: Linear

Meta-analysis type: Random-effects model

iteration | estimate Tn # to trim diff

----------+--------------------------------------

1 | 0.570 20 0 91

2 | 0.570 20 0 0

Note: no trimming performed; data unchanged
