# Supplementary material for: Efficacy and safety of sugammadex for neuromuscular blockade reversal in pediatric patients: an updated meta-analysis of randomized controlled trials with trial sequential analysis
Source: BMC Pediatr. 2022 May 19;22:295. doi: 10.1186/s12887-022-03288-0 (PMC9118813; doi:10.1186/s12887-022-03288-0)
Supplement: Supplementary file 3 — Additional file 3: Table S1. GRADE summary of findings table. [file 12887_2022_3288_MOESM3_ESM.docx]

| **TABLE S1. GRADE summary of findings table** | | | | | | | | | | | |
| --- | --- | --- | --- | --- | --- | --- | --- | --- | --- | --- | --- |
| **Quality assessment** | | | | | | | **Summary of Findings** | | | | |
| **Participants (studies) Follow up** | **Risk of bias** | **Inconsistency** | **Indirectness** | **Imprecision** | **Publication bias** | **Overall quality of evidence** | **Study event rates (%)** | | **Relative effect** (95% CI) | **Anticipated absolute effects** | |
|  |  |  |  |  |  |  | **With CONTROL** | **With SUG** |  | **Risk**  **with CONTROL** | **Risk difference with SUG** (95% CI) |
| **Primary outcomes** | | | | | | | | | | | |
| **Time to TOF>0.9 (CRITICAL OUTCOME)** | | | | | | | | | | | |
| 995 (17 studies) | no serious risk of bias | serious^1^ | no serious indirectness | no serious imprecision | reporting bias strongly suspected ^2^ | **⊕⊕OO** **LOW**^1,2^ due to inconsistency, publication bias | 479 | 516 | **-** |  | The mean time to tof>0.9 in the intervention groups was **14.42 lower** (17.08 to 11.75 lower) |
| **Extubation time (CRITICAL OUTCOME)** | | | | | | | | | | | |
| 883 (14 studies) | no serious risk of bias | serious^1^ | no serious indirectness | no serious imprecision | reporting bias strongly suspected ^2^ | **⊕⊕OO** **LOW**^1,2^ due to inconsistency, publication bias | 443 | 440 | **-** |  | The mean extubation time in the intervention groups was **13.98 lower** (16.7 to 11.26 lower) |
| **Secondary outcomes** | | | | | | | | | | | |
| **PONV (IMPORTANT OUTCOME)** | | | | | | | | | | | |
| 824 (13 studies) | no serious risk of bias | no serious inconsistency | no serious indirectness | no serious imprecision | undetected | **⊕⊕⊕⊕** **HIGH** | 69/393  (17.6%) | 33/431  (7.7%) | **RR 0.3**  (0.2 to 0.46) | **Study population** | |
|  |  |  |  |  |  |  |  |  |  | **176 per 1000** | **123 fewer per 1000** (from 95 fewer to 140 fewer) |
|  |  |  |  |  |  |  |  |  |  | **Moderate** | |
|  |  |  |  |  |  |  |  |  |  | **167 per 1000** | **117 fewer per 1000** (from 90 fewer to 134 fewer) |
| **Bradycardia (IMPORTANT OUTCOME)** | | | | | | | | | | | |
| 246 (4 studies) | no serious risk of bias | no serious inconsistency | no serious indirectness | very serious^3^ | undetected | **⊕⊕OO** **LOW**^3^ due to imprecision | 15/122  (12.3%) | 0/124  (0%) | **RR 0.09**  (0.02 to 0.46) | **Study population** | |
|  |  |  |  |  |  |  |  |  |  | **123 per 1000** | **112 fewer per 1000** (from 66 fewer to 120 fewer) |
|  |  |  |  |  |  |  |  |  |  | **Moderate** | |
|  |  |  |  |  |  |  |  |  |  | **117 per 1000** | **106 fewer per 1000** (from 63 fewer to 115 fewer) |
| **Pain (IMPORTANT OUTCOME)** | | | | | | | | | | | |
| 98 (2 studies) | no serious risk of bias | no serious inconsistency | no serious indirectness | very serious^3^ | undetected | **⊕⊕OO** **LOW**^3^ due to imprecision | 5/31  (16.1%) | 8/67  (11.9%) | **RR 1.21**  (0.46 to 3.17) | **Study population** | |
|  |  |  |  |  |  |  |  |  |  | **161 per 1000** | **34 more per 1000** (from 87 fewer to 350 more) |
|  |  |  |  |  |  |  |  |  |  | **Moderate** | |
|  |  |  |  |  |  |  |  |  |  | **125 per 1000** | **26 more per 1000** (from 67 fewer to 271 more) |
| **Bronchospasm/Laryngospasm (IMPORTANT OUTCOME)** | | | | | | | | | | | |
| 226 (3 studies) | no serious risk of bias | no serious inconsistency | no serious indirectness | very serious^3^ | undetected | **⊕⊕OO** **LOW**^3^ due to imprecision | 4/112  (3.6%) | 1/114  (0.9%) | **RR 0.45**  (0.1 to 1.96) | **Study population** | |
|  |  |  |  |  |  |  |  |  |  | **36 per 1000** | **20 fewer per 1000** (from 32 fewer to 34 more) |
|  |  |  |  |  |  |  |  |  |  | **Moderate** | |
|  |  |  |  |  |  |  |  |  |  | **43 per 1000** | **24 fewer per 1000** (from 39 fewer to 41 more) |
| **Dry mouth (IMPORTANT OUTCOME)** | | | | | | | | | | | |
| 120 (2 studies) | no serious risk of bias | no serious inconsistency | no serious indirectness | very serious^3^ | undetected | **⊕⊕OO** **LOW**^3^ due to imprecision | 25/60  (41.7%) | 3/60  (5%) | **RR 0.14**  (0.05 to 0.38) | **Study population** | |
|  |  |  |  |  |  |  |  |  |  | **417 per 1000** | **358 fewer per 1000** (from 258 fewer to 396 fewer) |
|  |  |  |  |  |  |  |  |  |  | **Moderate** | |
|  |  |  |  |  |  |  |  |  |  | **417 per 1000** | **359 fewer per 1000** (from 259 fewer to 396 fewer) |
| **Apnea (IMPORTANT OUTCOME)** | | | | | | | | | | | |
| 130 (2 studies) | no serious risk of bias | no serious inconsistency | no serious indirectness | very serious^3^ | undetected | **⊕⊕OO** **LOW**^3^ due to imprecision | 2/65  (3.1%) | 0/65  (0%) | **RR 0.33**  (0.04 to 3.12) | **Study population** | |
|  |  |  |  |  |  |  |  |  |  | **31 per 1000** | **21 fewer per 1000** (from 30 fewer to 65 more) |
|  |  |  |  |  |  |  |  |  |  | **Moderate** | |
|  |  |  |  |  |  |  |  |  |  | **31 per 1000** | **21 fewer per 1000** (from 30 fewer to 66 more) |
| **Oxygen desaturation (IMPORTANT OUTCOME)** | | | | | | | | | | | |
| 190 (3 studies) | no serious risk of bias | no serious inconsistency | no serious indirectness | very serious^3^ | undetected | **⊕⊕OO** **LOW**^3^ due to imprecision | 8/95  (8.4%) | 3/95  (3.2%) | **RR 0.41**  (0.12 to 1.37) | **Study population** | |
|  |  |  |  |  |  |  |  |  |  | **84 per 1000** | **50 fewer per 1000** (from 74 fewer to 31 more) |
|  |  |  |  |  |  |  |  |  |  | **Moderate** | |
|  |  |  |  |  |  |  |  |  |  | **57 per 1000** | **34 fewer per 1000** (from 50 fewer to 21 more) |

^1^ *I*^2^ > 50%
^2^ Publication bias was existed
^3^ Total number of events is less than 300
